# Measuring the Effect of Smoking on Hearing and Tinnitus Among the Adult Population in the Kingdom of Saudi Arabia

**DOI:** 10.7759/cureus.39689

**Published:** 2023-05-30

**Authors:** Mohahmmed Alateeq, Osama Alnizari, Tamara A Hafiz

**Affiliations:** 1 Otolaryngology-Head and Neck Surgery, University of Hail College of Medicine, Hail, SAU; 2 Family Medicine, University of Hail College of Medicine, Hail, SAU; 3 Public Health and Health Informatics, Umm Al-Qura University, Makkah, SAU

**Keywords:** tabaco, saudi arabia, tinnitus, hearing impairment, smoking

## Abstract

Background: The prevalence of cigarette smoking is a significant public health challenge in Saudi Arabia, as it is a known risk factor for many health problems. Hearing problems are also a major concern, as they are invisible disabilities that can negatively impact an individual's perception, communication, and social interactions. Studies have identified various risk factors for hearing loss, including genetics, diseases, infection, exposure to noise, and demographic factors such as age and gender. Smoking has been found to be associated with hearing loss, tinnitus, and vertigo, but the results of studies investigating this association have been inconsistent. It is crucial to understand the impact of smoking on hearing problems and tinnitus in the Saudi Arabian population to protect individual and societal health.

Aim: We aim to investigate whether smoking is related to tinnitus, hearing loss, or other hearing difficulties.

Methods: A cross-sectional study was established from March to August 2022 among adults in the Kingdom of Saudi Arabia to determine whether smoking can affect hearing.

Results: It has been observed that smokers experience hearing issues or trouble hearing more frequently than non-smokers. Additionally, as the number of cigarette smoking grows or as smoking persists for longer periods of time, there is a rise in hearing problems/hearing difficulty. In contrast, there is no conclusive evidence associating smoking and tinnitus.

Conclusion: These results should encourage more investigation into the impact of demographic factors on hearing problems/hearing difficulty, or tinnitus.

## Introduction

Cigarette smoking is a major public health challenge in the Kingdom of Saudi Arabia, as it is a prevalent lifestyle factor and a well-known risk factor for various health issues [1]. Besides that, hearing problems and disorders are prevalent and often invisible disabilities that can have a profound impact on individuals' lives and society as a whole. When left unmanaged and untreated, hearing problems can impair perception, communication, and speech, leading to social isolation, loneliness, and stigma. Additionally, the psychological pressure experienced by those with hearing impairments due to difficulties in communication can be one of the greatest sources of psychological distress for individuals [2,3]. The World Health Organization has applauded predictions that by 2050, 2.5 billion people worldwide would experience hearing issues, such as hearing loss, and that there will be greater demand for hearing rehabilitation services [3].

Research has indicated that different risk factors for hearing loss may be genetic or related to complications or negative consequences of diseases, infections, the use of ototoxic drugs, exposure to noise, etc. In addition, several studies have explored the impact of demographic factors such as gender, aging, and smoking on hearing loss [4,5]. Age-related hearing loss typically begins in the third decade of life and develops gradually [6], and men are reported to be more susceptible to hearing loss [5,7]. Moreover, studies have shown that both aging and smoking can double the incidence of hearing loss [8]. Elderly individuals who smoke cigarettes for extended periods may experience reduced blood circulation in the cochlea, leading to an increased risk of hearing loss [9].

Therefore, it is essential to investigate the relationship between smoking and hearing problems while paying attention to demographic factors such as age and gender, as well as smoking habits, the duration of time, and the number of cigarettes smoked per day. A study revealed that women who have smoked in the past or present are more likely to suffer from moderate or severe hearing loss, with the magnitude of risk increasing with the number of years smoked [10]. Studies confirmed that smokers of cigarettes are more likely to have hearing loss, tinnitus, and vertigo [1]. Other studies, in a meta-analysis, found a statistically significant relationship between smoking and tinnitus [11]. Tinnitus is a condition defined as the feeling of hearing sounds without being able to distinguish their source [11] or a condition defined as hearing sounds that are not produced by the outside world [12], and although there is a cumulative body of research showing that smoking causes hearing problems, the results of studies exploring the association between smoking and hearing problems have been inconsistent [9].

Considering the impact of smoking and hearing problems, including tinnitus, on the quality of life of individuals and society, it is crucial to reveal the effect of smoking on hearing problems or tinnitus in the general population of the Kingdom of Saudi Arabia to safeguard people's health. Thus, a comprehensive understanding of the relationship between smoking and hearing problems is necessary to develop effective strategies to prevent and manage these conditions. In addition, according to the researchers' knowledge, there is a lack of studies conducted in Saudi Arabia that aim to measure the relationship between smoking and hearing, as well as tinnitus. Therefore, this study aims to measure the prevalence of smoking, hearing problems, and tinnitus and also assess the effect of smoking on hearing, or tinnitus, as well as investigate the potential role of covariates (sociodemographic factors and chronic diseases) as determinants of risk for hearing problems or tinnitus, among a large population sample, 18-60 years of age, who represents that population group at the national level.

## Materials and methods

A cross-sectional study was conducted from March to August 2022 to assess the effect of smoking on hearing among the adult population of Saudi Arabia.

The sample size was calculated using the Raosoft sample size calculator, taking into account a total adult population of 25,777,851 (aged 18-60), an expected response rate of 50%, a margin of error of 5%, and a standard deviation of 1.96 for the 99% confidence interval (CI). According to the General Authority for Statistics' yearly statistics for the current mid-2020s, a minimum sample size of 664 was required. We doubled the sample size to 1,328 and added a 65% increase to create a more representative sample of survey participants and decrease bias. After applying the exclusion criteria, 2,209 participants from the western, northern, southern, central, and eastern parts of the Kingdom participated in the study.

The inclusion criteria were the general adult population, ages 18-60, in all administrative districts within the Kingdom, while participants under the age of 18 and over the age of 60 were excluded. The questionnaire was self-administered and took between five and eight minutes to complete, consisting of 15 items categorized as follows: (1) sociodemographic characteristics, including age, gender, residency region, nationality, marital status, occupation, and level of education; (2) smoking status and presence of chronic diseases; (3) Hearing Handicap Inventory Screening Questionnaire for Adults; and (4) Tinnitus Handicap Inventory (THI).

Statistical analyses were conducted using Statistical Package for the Social Sciences (SPSS) version 25.0 (IBM SPSS Statistics, Armonk, NY, USA). Descriptive statistics, including frequency and proportions, were used to analyze all quantitative variables, including demographic characteristics. Chi-square tests were performed to determine the association between the smoking index and hearing problems/difficulty and tinnitus, as well as to evaluate the demographic characteristics related to these conditions. Logistic regression analysis was also used to predict the presence of hearing problems/difficulty and tinnitus (dependent variable), where odds ratios (OR) and 95% confidence intervals (CIs) were calculated for each independent variable. The statistical significance level was set at p < 0.05, and the results were presented using tables and graphs.

The study was granted ethical approval with the number H-2022-310 by the Hail University Medical Research Ethics Committee on December 9, 2022. Before participating, every participant received the questionnaire with their explicit permission and consent, and any ethical concerns were addressed beforehand.

## Results

A total of 1,939 individuals in the Kingdom of Saudi Arabia were enrolled in the study, including 1,061 men and 878 women, with ages ranging from 18 to 60. The majority (55.2%) were between the ages of 18 and 30. Of those who participated in the study, 1,014 (45.9%) were smokers, while 308 (13.5%) used e-cigarettes. The remaining 617 (31.9%) were non-smokers (Table 1).

**Table 1:** General characteristics of the studied sample. ^1^The number of people with diabetes was 122 (5.3%), hypertension was 166 (7.3%), asthma was 140 (6.1%), high cholesterol was 96 (4.2%), kidney disease was 58 (2.5%), cardiovascular disease was 43 (1.9%), mental/psychological illness was 36 (1.6%), and cancer was four (0.2%).

Variables		N = 2,209	%
Age (years)	18-30 years	1,219	55.2
	31-40 years	574	26
	41-50 years	302	13.7
	51-60 years	114	5.2
Gender	Male	1,331	60.3
	Female	878	39.7
Marital status	Married	1,061	48
	Unmarried	1,148	52
Nationality	Saudi	2,058	93.2
	Non-Saudi	151	6.8
Region	Western	534	23.3
	Central	551	24.1
	Eastern	395	17.3
	Southern	453	19.8
	Northern	355	15.5
Educational level	Primary school	40	1.8
	Intermediate school	76	3.4
	High school/diploma	806	36.5
	Bachelor’s degree	1,194	54.1
	Master’s degree	93	4.2
Occupation	Governmental sector	617	27.9
	Private sector	540	24.4
	Retired	86	3.9
	Student	558	25.3
	Do not work	408	18.5
Chronic diseases	Yes^1^	634	28.7
	No	1,575	71.3
Smoking habit	Current smoker	1,014	45.9
	Ex-smoker	287	13
	Non-smoker	908	41.1
How many cigarettes per day?	More than 20	438	19.8
	Less than 20	500	22.6
	I use electronic cigarette	308	13.5
How long have you been smoking?	More than five years	771	34.9
	Less than five years	390	17.7
	I recently quit	136	6.2

**Figure 1:**
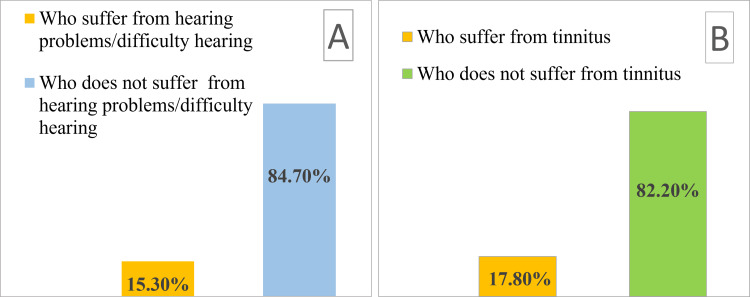
Distribution of individuals suffering from hearing problems, difficulty hearing, and tinnitus. A: Distribution of hearing problems and difficulty. B: Distribution of tinnitus.

Figure 1 shows the distribution of participants who reported hearing problems or difficulty hearing and tinnitus. As shown in Figure 1A, a total of 1,871 (84.7%) participants did not report hearing problems or difficulty hearing, while 338 (15.3%) participants reported hearing problems or difficulty hearing. Similarly, in Figure 1B, a total of 1,816 (82.2%) participants did not report tinnitus, while 393 (17.8%) participants reported tinnitus.

**Figure 2:**
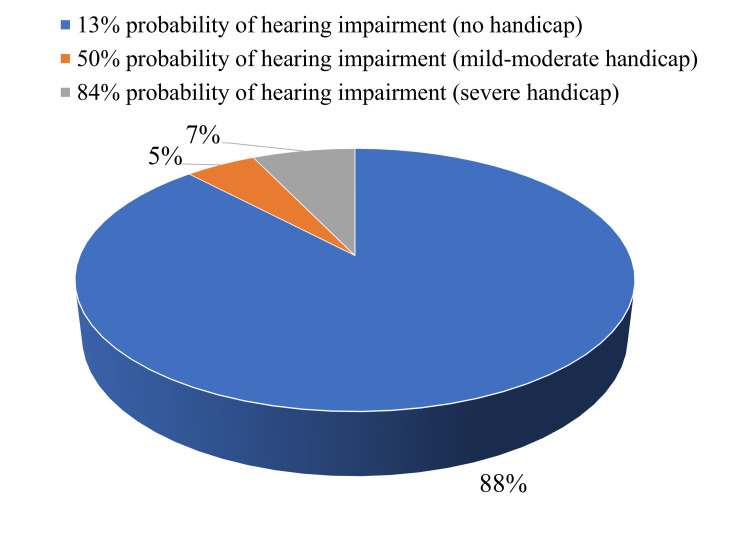
Hearing problems/difficulty hearing among adults according to the Hearing Handicap Inventory Screening Questionnaire for Adults.

The results of the screening of the Hearing Handicap Inventory indicate that most participants, or 1,944 participants, did not have a hearing impairment and had a 13% probability of experiencing difficulty in hearing. However, 109 (4.9%) participants had mild to moderate hearing impairments with a 50% likelihood of experiencing hearing difficulties, and 156 (7.1%) participants had severe hearing impairments with an 84% probability of experiencing hearing difficulties, as shown in Figure 2.

**Figure 3:**
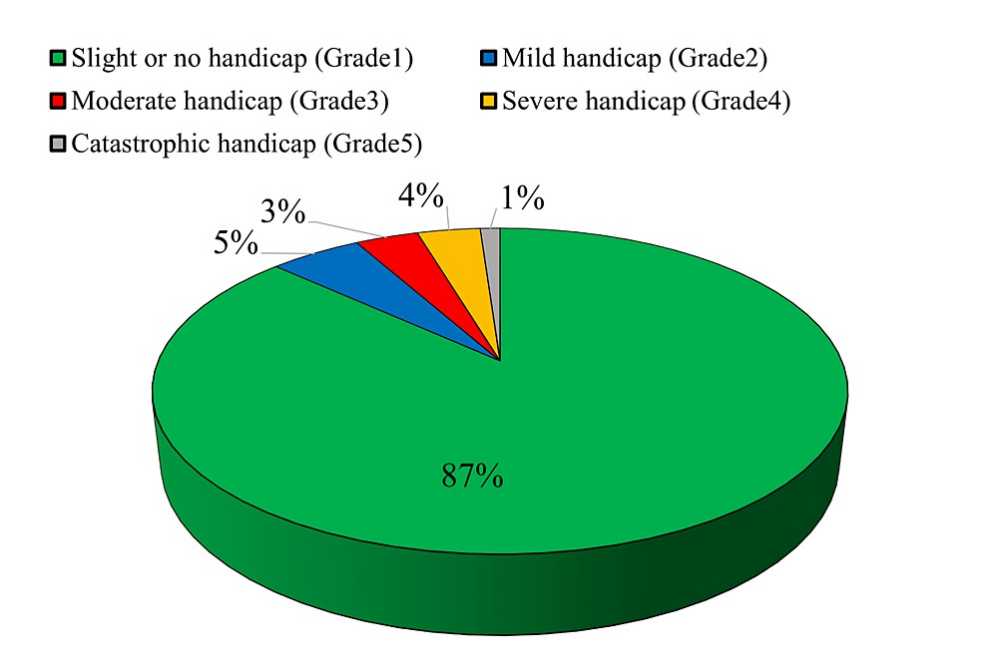
Tinnitus among adults according to the THI. THI: Tinnitus Handicap Inventory

According to the results of the Tinnitus Handicap Inventory shown in Figure 3, the majority of participants had a slight or no handicap (Grade 1), while 113 (5.1%) had a mild handicap (Grade 2), and 77 (3.5%) and 78 (3.5%) had a moderate (Grade 3) and severe handicap (Grade 4), respectively. Participants who had a catastrophic handicap (Grade 5) constituted the lowest percentage of the participants at 25 (1.1%).

Investigation of hearing problems/difficulty of hearing

When considering the influence of social and demographic factors on the occurrence of hearing problems/difficulty of hearing, the majority of individuals did not express concern about hearing issues or difficulties; however, 338 (15.3%) participants did (Figure 1A).

After comparing the ages of participants who experienced hearing problems/difficulty hearing, the majority of those were in the age range of 18-30 years, while 4.9% were aged 31-40 years, and 2.3% were aged 41-50 years. The age group of 51 years and over had the lowest percentage of participants who experienced hearing problems/difficulty hearing at 1.4%. There was a significant association between age and hearing problems/difficulty hearing (p = 0.000), as the majority of individuals with these issues were in the age group of 18-30 years.

As for gender differences, participating males experienced greater hearing problems/difficulty than participating females, and there was a significant association between gender and suffering from hearing problems/difficulty hearing (p = 0.000). Regarding marital status, there was a significant association between marital status and hearing problems/difficulty (p = 0.000), with 4.9% of unmarried participants and 10.4% of married participants experiencing hearing problems/difficulty hearing, but married people experience these issues more frequently. There was no significant association between Saudi and non-Saudi participants with hearing problems/difficulty (p = 0.467). However, there was a significant association (p = 0.000) between regional differences in Saudi Arabia and hearing problems/difficulty hearing, as individuals in the southern region had a higher risk of experiencing these issues.

The majority of individuals with hearing problems/difficulties of hearing were found to be in high school/diploma and bachelor's stages, indicating a significant association between education level and these conditions (p = 0.000). When comparing occupation and the presence of hearing problems/difficulty hearing, it was found that both public and private sector workers suffered from the presence of these issues, with a higher frequency among workers in the private sector. This indicates a significant association between occupation and hearing problems/difficulty hearing (p = 0.000).

Similarly, when comparing participants who had chronic diseases with those who did not, an association was observed between the presence of chronic diseases and the occurrence of hearing problems/difficulty hearing (p = 0.000) (Table 2).

**Table 2:** Comparison of sociodemographic characteristics of participants with hearing problems/difficulty of hearing or tinnitus with participants without hearing problems/difficulty of hearing or tinnitus. *Significant

Variables		With hearing problems (number (%))	Without hearing problems (number (%))	P value	With tinnitus (number (%))	Without tinnitus (number (%))	P value
Age (years)	18-30 years	150 (6.8)	1,069 (48.4)	0.000*	203 (9.2)	1,016 (46)	0.115
	31-40 years	108 (4.9)	466 (21.1)		99 (4.5)	475 (21.5)	
	41-50 years	50 (2.3)	252 (11.4)		66 (3)	236 (10.7)	
	51-60 years	30 (1.4)	84 (3.8)		25 (1.1)	89 (4)	
Gender	Male	256 (11.6)	1,075 (48.7)	0.000*	216 (9.8)	1,115 (50.5)	0.018*
	Female	82 (3.7)	796 (36)		177 (8)	701 (31.7)	
Marital status	Single	109 (4.9)	952 (43.1)	0.000*	174 (7.9)	887 (40.2)	0.100
	Married	229 (10.4)	919 (41.6)		219 (9.9)	929 (42.1)	
Nationality	Saudi	318 (14.4)	1,740 (78.8)	0.467	366 (16.6)	1,692 (76.6)	0.976
	Non-Saudi	20 (0.9)	131 (5.9)		27 (1.2)	124 (5.6)	
Region	Western	56 (2.5)	468 (21.2)	0.000*	78 (3.5)	446 (20.2)	0.000*
	Eastern	47 (2.1)	319 (14.4)		70 (3.2)	296 (13.4)	
	Northern	36 (1.6)	309 (14)		78 (3.5)	267 (12.1)	
	Southern	159 (7.2)	280 (12.7)		98 (4.4)	341 (15.4)	
	Central	40 (9.4)	495 (22.4)		69 (3.1)	466 (21.1)	
Educational level	Primary school	15 (0.7)	25 (1.1)	0.000*	9 (0.4)	31 (1.4)	0.760
	Intermediate school	23 (1.0)	53 (2.4)		13 (0.6)	63 (2.9)	
	High school/diploma	157 (7.1)	649 (29.4)		137 (6.2)	669 (30.3)	
	Bachelor’s degree	133 (6)	1,061 (48)		214 (9.7)	980 (44.4)	
	Master’s degree	10 (0.5)	83 (3.8)		20 (0.9)	73 (3.3)	
Occupation	Do not work	49 (2.2)	359 (16.3)	0.000*	71 (3.2)	337 (15.3)	0.019*
	Retired	28 (1.3)	58 (2.6)		27 (1.2)	59 (2.7)	
	Student	56 (2.5)	502 (22.7)		97 (4.4)	461 (20.9)	
	Private sector	121 (5.5)	419 (19)		88 (4)	452 (20.5)	
	Governmental sector	84 (3.8)	533 (24.1)		1,109 (5)	507 (23)	
Chronic diseases	Yes	211 (9.6)	423 (19.1)	0.000*	153 (6.9)	481 (21.8)	0.000*
	No	127 (5.7)	1,448 (65.6)		240 (10.9)	1,335 (60.4)	

When investigating the relationship between smoking and its specific factors as risk factors for hearing problems/difficulty hearing, it was found that there is a significant association between smoking and suffering from hearing problems/difficulty hearing (p = 0.000). Smokers were observed to suffer from hearing problems/difficulty hearing more frequently than non-smokers. Additionally, smokers who smoke more than 20 cigarettes a day have a higher frequency of hearing problems/difficulty hearing than those who smoke less than that or use e-cigarettes. Similarly, smokers who have smoked cigarettes for more than five years have a higher frequency of hearing problems/difficulty hearing than those who have smoked recently or for less than five years. This indicates that as the number of cigarettes smoked increases, there is an increase in hearing problems/hard of hearing, and if cigarette smoking continues for a longer period of time, the frequency of hearing problems/difficulty hearing also increases (Table 3).

**Table 3:** Comparison of smoking habits of participants with hearing problems/difficulty of hearing or tinnitus with participants without hearing problems/difficulty of hearing or tinnitus. *Significant

Variables		With hearing problems (number (%))	Without hearing problems (number (%))		P value	With tinnitus (number (%))	Without tinnitus (number (%))	P value
Smoking habit	Non-smoker	63 (2.9)	845 (38.3)	0.000*		153 (6.9)	755 (34.2)	0.627
	Smoker	221 (10)	793 (35.9)			187 (8.5)	827 (37.4)	
	Ex-smoker	54 (2.4)	233 (10.5)			53 (2.4)	234 (10.6)	
How many cigarettes per day?	More than 20	125 (5.7)	313 (14.2)	0.000*		85 (3.8)	353 (16)	0.275
	Less than 20	74 (3.3)	426 (19.3)			82 (3.7)	418 (18.9)	
	Use e-cigarette	64 (2.9)	234 (10.6)			62 (2.8)	236 (10.7)	
How long have you been smoking?	More than five years	187 (8.5)	584 (26.4)	0.000*		156 (7.1)	615 (27.8)	0.089
	Less than five years	61 (2.8)	329 (14.9)			57 (2.6)	333 (15.1)	
	I recently quit	26 (1.2)	110 (5)			26 (1.2)	110 (5)	

Investigation of tinnitus

When considering the influence of sociodemographic factors on the incidence of tinnitus, the majority of individuals did not express that they had tinnitus, but 393 (17.8%) participants did (Figure 1B).

After comparing the ages of participants with tinnitus, the majority of those were in the age group of 18-30 years (9.2%), followed by 4.5% in the age group of 31-40 years, and 3% in the age group of 41-50 years. The age group of 51 years and over had the lowest percentage of participants who reported experiencing tinnitus at 1.1%. However, there was no significant association between age and tinnitus (p = 0.115).

Regarding gender differences, male participants were observed to suffer from tinnitus more frequently than female participants, and there was a significant association between gender and suffering from tinnitus (p = 0.018). As for differences in marital status, there was no significant association between marital status and tinnitus (p = 0.100). Similarly, there was no significant association between Saudi and non-Saudi participants with tinnitus (p = 0.976). However, there was a significant association (p = 0.000) between regional differences in Saudi Arabia and tinnitus, with individuals in the southern region having a higher risk of developing these problems, despite the proportions being similar in all regions of the Kingdom.

Regarding educational level and its relationship to tinnitus, there was no significant association between educational level and tinnitus (p = 0.760). On the contrary, in terms of the difference in occupation and the presence of tinnitus, it was found that students were more likely to suffer from tinnitus compared to workers, retirees, and non-workers. This indicates an association between occupation and tinnitus (p = 0.019).

When comparing participants who suffered from chronic diseases with those who did not but suffered from tinnitus, an association was observed between chronic diseases and tinnitus (p = 0.000). Participants who did not suffer from chronic diseases were found to be the most vulnerable group to tinnitus (Table 2).

When investigating the relationship between smoking and its factors specifically, as risk factors for tinnitus, there is no significant association (Table 3).

When using binary logistic regression to predict smoking habit as an independent variable associated with hearing problems/difficulty hearing, it was found that 221 (10%) smokers suffered from hearing problems/difficulty hearing. The odds ratio and 95% confidence interval for hearing problems/difficulty hearing in smokers were 0.908 (0.886-0.929) (p < 0.05), indicating that smoking was a significant predictor of hearing problems/difficulty hearing in smokers (Table 4).

**Table 4:** The effect of any tobacco use on the development of hearing problems/difficulty of hearing or tinnitus. OR: odds ratio, AOR: adjusted odds ratio, CI: confidence interval Dependent variables: suffering from hearing problems/difficulty Predictors (constant): smoker status *Significant

Predictor	Total (N = 2,209)	Smoking (n = 1,014)	Non-smoking (n = 908)	OR (95% CI)	P value
Hearing problems/difficulty	338	221 (10)	63 (2.9)	0.908 (0.886-0.929)	0.000*

## Discussion

Our study's goals are to determine the prevalence of smoking habits along with each hearing problem/hearing difficulty and tinnitus, as well as investigate how smoking habits affect these conditions.

Smoking in the Kingdom of Saudi Arabia

Upon considering the prevalence of smoking, our study found a significant prevalence of smoking in Saudi society, with almost half of the participants (1,014 individuals) being current smokers, resulting in a 45.9% smoking rate. Additionally, 13% of the participants were ex-smokers. This indicates an increase in smoking habits compared to a previous local survey, which showed a smoking prevalence of 12.8% among the Saudi population [13]. Another study in Riyadh found that 19.5% of people currently smoke, with 42.8% being ex-smokers [14]. Additionally, Al-Nozha et al. [13] found that 17.6% of individuals were current smokers and 39.8% were ex-smokers. Since each of those two studies revealed an increase in the proportion of ex-smokers relative to the proportion of current smokers, the results of our study contradict each of those two studies in light of this. Furthermore, our research showed that smoking prevalence was higher in the southern portion of Saudi Arabia compared to the other regions, among males more so than females, between the ages of 18 and 30, in comparison to other age groups. Similar results were found by Al-Nozha et al. [13], who during a five-year period performed a survey on smoking among Saudi adults in all parts of the country between the ages of 30 and 70. Men smoked significantly more than women did [15]. This study contradicts our findings on smoking prevalence's geographical distribution. Smoking is more common among Saudis living in urban, western, northern, and eastern regions than in other parts of the country.

Hearing problems/difficulty hearing in the Kingdom of Saudi Arabia

First, upon considering the prevalence of hearing problems/difficulty of hearing, our study found that hearing problems are not as prevalent in Saudi society as smoking is.

However, this study found a significant association between smoking and hearing issues such as difficulty hearing and hearing loss. This association has been reported in several other studies [16-21]. It is important to note that some studies, including a comparative study [22] and a cohort study by Gates et al. [23], did not find a connection between smoking and hearing loss.

Second, when investigating the role of sociodemographic factors in hearing problems of hearing, this study found a significant association between sociodemographic factors and hearing difficulties. Age, gender, marital status, region, education, occupation, and chronic illness were all linked to hearing problems. Specifically, married, middle-aged men from the southern region with a bachelor's or diploma degree, working in the private sector, and with chronic diseases were more likely to have hearing issues. The National Institute on Deafness and Other Communication Disorders also reported that men are more likely to experience hearing problems than women [24]. In addition to a prior study measuring the prevalence of hearing problems in Saudi patients with type 2 diabetes, which revealed a strong association between type 2 diabetes mellitus and hearing loss, it was found that hearing loss was found to be more common in people with diabetic problems, and insulin control of blood sugar levels was found to increase the risk of hearing loss [25]. Our findings also support the connection between chronic diseases and hearing problems. Therefore, it is crucial to investigate prevalent chronic conditions such as hypertension and diabetes separately to better understand their impact on hearing.

Tinnitus in the Kingdom of Saudi Arabia

Upon considering the prevalence of tinnitus, the findings of our study revealed a non-significant prevalence of tinnitus in Saudi society, in the same context as hearing problems.

Our investigation found no significant association between smoking and tinnitus. However, we did find that gender, regional differences, educational level, chronic disease, and suffering from tinnitus were significantly associated. Specifically, males, students, participants from the southern region, and those with chronic diseases were more likely to suffer from tinnitus. A previous study revealed that males had higher disability measures than females, particularly in emotional and functional measurements, with 76% of patients experiencing tinnitus-related hearing loss. Male patients also experienced higher severity scores for the disease, 15.3 points higher than female patients [26]. In contrast, another study found no significant association between gender, age, and the severity of tinnitus [27].

To the best of our knowledge, this was the first study to investigate the association between smoking, tinnitus, and hearing problems in Saudi Arabia. However, our study was limited by its cross-sectional design, which may have resulted in retrieval inaccuracies and imprecise results. Additionally, we excluded pediatric and senior age groups to focus on specific age ranges. Therefore, we recommend further experimental and prospective research with larger, multicenter samples to better understand the link between smoking, tinnitus, and hearing issues.

## Conclusions

The study found that there is an association between smoking and hearing problems, and unlike tinnitus, there was no clear evidence of an association. The results also indicated a significant influence of demographic factors, as these factors showed differences in the hearing health status of smokers compared to non-smokers.

Based on the conclusion, several recommendations can be made to further investigate the link between smoking and hearing problems. Firstly, more studies should be conducted to determine the specific types of hearing problems associated with smoking, such as reduced hearing sensitivity or difficulty understanding speech. Secondly, demographic factors that impact hearing problems and tinnitus should be further specified, with larger sample sizes and more detailed data collection methods. Thirdly, health awareness and promotion specialists should develop targeted educational materials and resources for patients who smoke or have hearing problems to highlight the link between smoking and hearing problems. Fourthly, ear, nose, and throat (ENT) doctors should routinely screen patients for smoking and hearing problems and provide smoking cessation counseling as part of their practice. Fifthly, awareness campaigns and smoking cessation sessions should be tailored to specific populations, such as adolescents or pregnant women, who may be more vulnerable to the negative effects of smoking on hearing. Lastly, longitudinal studies should be conducted to determine whether quitting smoking can improve hearing outcomes over time. By following these recommendations, it is hoped that a better understanding of the link between smoking and hearing problems can be established, and effective interventions can be developed to improve hearing outcomes in smokers.
